# Evaluation of 5536 patients treated in an integrative outpatient tinnitus treatment center–immediate effects and a modeling approach for sustainability

**DOI:** 10.1186/s12913-016-1644-7

**Published:** 2016-08-11

**Authors:** Thomas Ostermann, Katja Boehm, Martin Kusatz

**Affiliations:** 1 Faculty of Health, Department of Psychology and Psychotherapy, Chair of Research Methodology and Statistics in Psychology, Witten/Herdecke University, 58448 Witten, Germany; 2 Tinnitus Therapy Center (TTZ), Krefeld-Düsseldorf, Krefeld, Germany

**Keywords:** Tinnitus, Out-patients, Multiple imputation, Cohort-study, Regression to the mean

## Abstract

**Background:**

Tinnitus is an increasingly serious problem for health care systems. According to epidemiological data, 7–14 % of outpatients have asked their physician about tinnitus and management strategies. Integrative outpatient treatments are currently regarded as promising therapeutic approaches for managing tinnitus. In this article we report on the treatment success of an outpatient tinnitus treatment center in Germany.

**Methods:**

This cohort study included pre-post data of 5536 outpatients which were treated between 2003 and 2010 in the tinnitus-therapy center, Krefeld-Düsseldorf (TTZ). The intervention consisted of psychological immunization training as well as an auditory stimulation therapy component. The main outcome parameter was the score of the Tinnitus Questionnaire (TQ) which was assessed before and after a 9 days treatment and (in a small subsample) at a 6 months follow-up. Missing data were multiply imputed. Pre-post effect sizes were calculated and adjusted for regression to the mean (RTM).

**Results:**

RTM-adjusted treatment effects at the end of treatment were estimated as −18.6 (CI: −18.9 to 18.2, *p <* 0.001) score points which corresponds to a standardized effect of d = −1.03 (CI: −1.05 to −1.01). These effects can be corroborated in various subgroups and all subscales of the TQ (d ranging from −0.31 to −0.97).

**Conclusion:**

The study suggests the effectiveness of this outpatient tinnitus therapy concept. Multiple imputations techniques and RTM analysis were helpful in carving out true treatment effects.

## Background

Tinnitus is a major problem of almost all Western health care systems. It is often associated with sensorineural hearing impairment but the effects of tinnitus are primarily of psychosocial nature [1]. According to epidemiological studies in different countries the prevalence of tinnitus, varies between 4.4 and 15.1 % for adults and between 7.6 and 20.1 % for individuals below the age of 50 years [2]. A study in four English cities found that tinnitus occurred in 17.5 % of the participants in the age group of 40–60 years and 22.2 % in participants above the age of 60 years [3]. It is estimated that for 15–21 % of the adult population, tinnitus is a fairly stable auditory sensation, for a subgroup of 3–5 %, it becomes a bothersome and incapacitating symptom, seriously interfering with all aspects of daily life [4–6]. According to epidemiological data, 7–14 % of out-patients have asked their physician about tinnitus and management strategies [1].

About 1–2 % of the population is severely disturbed by tinnitus yielding to an impaired body function, concentration difficulties, absence from work, disruption of everyday activities and sleep. A decompensated tinnitus in most cases is accompanied by complaints like anxiety, depression, and a loss of quality of life. With regards to health economic aspects, a recent study from the Netherlands revealed that the economical burden of tinnitus to the society is quite substantial with the severity of tinnitus being an important predictor of costs created by patients [7].

Several treatments for chronic tinnitus have thus been proposed ranging from outpatient concepts to rehabilitation programs in specialized hospitals. Taking into account that tinnitus symptoms are widespread and individually different integrative approaches in treating tinnitus have been proposed. These include cognitive-behavioral treatment (CBT) [8], relaxation techniques [9] as well as sound therapeutic options to reduce the acoustic symptoms and are regarded as promising for managing tinnitus [10]. A recent review of 31 studies [11] found multidisciplinary CBT-based tinnitus treatment the most promising option for treating patients with tinnitus. Moreover, according to [12] “sound therapy should be considered an essential component of any clinical program of tinnitus management”. A recent survey investigating the ‘Heidelberg Model of Music Therapy’ in *N =* 206 chronic tinnitus patients with a follow-up time of 2.65 (SD 1.1) years suggests that such treatment models seems to be effective in the long run [13].

Most of these therapeutic concepts however still lack a substantial evidence base. A meta-analysis of Hesser et al. [14] investigating cognitive–behavioral therapy for tinnitus distress found 15 randomized controlled trials (RCTs) typically with a low to moderate study quality and a small or moderate sample size (mean sample size ± std-dev: 69 ± 33).

Apart from a low sample size another major problem in conduction high quality studies in tinnitus is to obtain data on long-term reductions of tinnitus severity. A recent evaluation found that a very high percentage of tinnitus patients did not return to the tinnitus clinic for follow-up visits. Thus no follow up data was obtained [15]. This is underpinned by the meta-analysis by Hoare et al. [16] on the efficacy of auditory perceptual training in the treatment of tinnitus: 7 of 10 included studies did not report follow-up data beyond the end of therapy. Moreover, half of the included studies suffered from a lack of a control group and only one study was blinded [16]. Thus, evidence on the effects of tinnitus therapies is mainly derived from uncontrolled studies with a small sample size and no or insufficient follow up data.

Structured data from quality management might help to produce valid and complete data in a high number of patients before and after therapy but is seldom used as an evidence base although a first attempt to use such data was already described in 1995 [17].

This paper reports the results from an evaluation of an outpatient tinnitus treatment of the Tinnitus Therapy Center Krefeld-Düsseldorf (Germany) based on quality management data including multiple imputation techniques and control for regression to the mean. The intervention consisted of psychological immunization training as well as an auditory stimulation therapy component. With this piece of research the authors want to evaluate an outpatient treatment based on quality management data. However, instead of conducting a controlled trial, it is rather aimed at “real world effectiveness”. This question is of great relevance, given the often small-scale controlled trials on tinnitus treatments, offering but limited ecological validity.

## Methods

### Ethical considerations

As this was a non-invasive, retrospective cohort study and all data analysed were collected as part of routine diagnosis and treatment which was not set up as a study or research project, but as a registered treatment program of the German Statutory Health Insurances there was no necessity to obtain a vote from a local research ethics committee [18]. However, the declation of Helsinki and the rules for data protection and data security and good epidemiological practice were fully applied. All patients gave verbal informed consent that their data could be used for scientific purposes.

### Sample recruitment

Patients consulting their general practitioner were referred to the tinnitus therapy center if a subacute or chronic tinnitus (duration > six months) was diagnosed and no psychiatric co-morbidity was given. All patients who were treated between 2003 and 2010 at the tinnitus therapy center, Krefeld-Duesseldorf (TTZ), Germany and completed the questionnaires were included in this evaluation.

### Intervention

The actual therapeutic approach consisted of a 9-day outpatient treatment including a total of 24 h of progressive muscle relaxation according to Jacobson and cognitive methods of restructuring and mindfulness-based techniques including aspects of counselling and emotional accompaniment, positive imagination techniques, attentional engagement and mental refocussing on inner resources. Patients are put in the position to manage the unpleasantness of tinnitus through active self-control. Furthermore, a defocusing of attention away from tinnitus is supported, thus raising the tolerance towards it.

This approach is complemented with 15 hours of Auditive Stimulation Therapy AST® which includes receptive psychoacoustic training, musical perception training and music therapeutic exercises originally employed in the treatment of chronic pain and developed specifically for tinnitus treatment [19]. In the institute’s own recording studio these programmes are being constantly updated and matched to the needs of the patients. Each patient receives a recording of these training programmes, so that even after ending the therapy she can work with them independently. This package aims at improving patients’ control of ear sounds i.e. by lowering the level of sensitivity to sounds and to relieve their feelings of helplessness. Furthermore, special exercises are learnt and carried out, on the one hand to increase self-control over the tinnitus and on the other hand to bring about a change in therapeutically unfavourable behaviour patterns. Thus, through the close-to-home outpatient treatment, the patient stays in his social environment, which reduces the rate of recurrence. Within this framework specialized physicians are available as consultants to answer questions and if necessary make therapy recommendations.

**Fig. 1:**
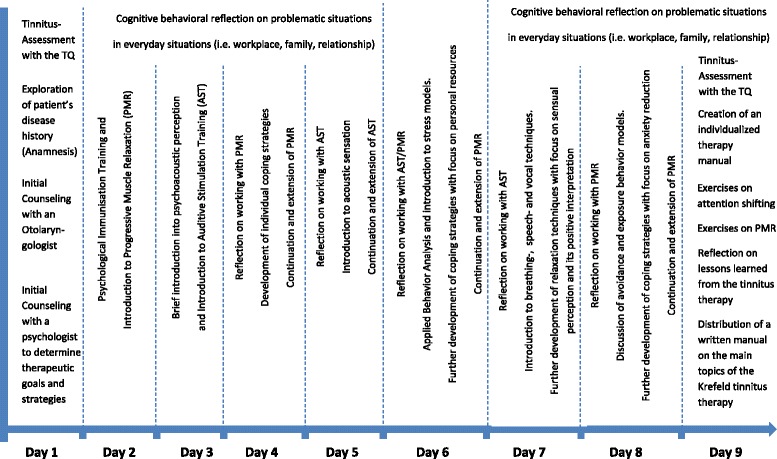
Scheme of the treatment

A detailed description of the complete program is given in Fig. 1 and in [20].

#### Outcome measures

For each imputed data set, treatment effects were estimated on the basis of the Tinnitus-Questionnaire (TF) developed by Goebel and Hiller [21]. This 42-item questionnaire has proven to be a valid and reliable outcome measure with corrected item-total correlations between 0.34 to 0.69 and Cronbach’s alpha of 0.95. Correlations to other tinnitus-related questionnaires were very high with r between 0.83 to 0.90 and change sensitivity and robustness against unchanged tinnitus conditions was also given [22].

The TQ covers a broad range of tinnitus-related and general psychological complaints in tinnitus patients by measuring a patient’s stress.

Patients respond to the items from “true”, “partly true” or “not true”. Responses could range from 0 points (no complaints at all) to a maximum of 84 points. The obtained total score provides a global level of severity and stress with the division in slight (up to 30 points), mediocre (31–46 points), severe stress (47–59 points) or extremely severe stress (60–84 points). Up to a total score of 46 the tinnitus is classified as ‘compensated’ and ≥ 47 as ‘decompensated’.

The 6 subscales measure the following dimensions:

E; Emotional Distress, Co; Cognitive Distress, InTi; Intrusiveness, Aku; Auditory Perceptual Difficulties, Sl; Sleep Disturbances and Som; Somatic Complaints. Both subscales ‘emotional stress’ and ‘cognitive stress‘ were assigned to the area ‘psychological stress (PB).

Although all subscales according to [23] have been shown to be convergent the “Sleep Disturbances” subscale in particular to has shown a high potential in determining specific effects of an intervention.

The TQ has been validated in different languages (i.e. Dutch, French and Chinese) and is the most widely used questionnaire in German-speaking areas as a primary outcome measurement in clinical trials [22].

#### Multiple imputation

As only sparse follow-up data was available, we used 6-month follow up data from *n =* 106 patients from the same center [19] as a basis to replace missing values by means of multiple imputations following the suggestions of Rubin [24]. Instead of filling in a single value as a substitute for a missing value, multiple imputation is a strategy by which each missing value is replaced simultaneously by a set of plausible values that represents the uncertainty about the right value to impute. Thus, each missing value is filled in several times generating several distinct data tables, each with a complete set of data relating to all patients without any missing values. These complete data tables are analysed separately using appropriate statistical models. Afterwards, the results from all statistical analyses are pooled to generate treatment effects and *p-*values.

With respect to the different types of missing data, multiple imputation is based on data missing at random (MAR). As in data missing completely at random (MCAR) the missingness does not depend on the values of any other variables of the study, we had to exclude some patients for multiple imputation, which results in the different numbers on *n*.

In our study we used the MCMC (Markov Chain Monte Carlo) replacement method based on logit transformed data according to the recommendations by Vroomen et al. [25] and Žliobaite et al. [26] for massively missing data and created 10 multiply imputed data tables. Both replacement of missing values and pooling of the results were done with the MI and MIANALYZE procedure of SAS 9.2 ® statistical software [27].

A more detailed description of multiple imputation and its assumptions and limitations is given in [28, 29].

#### Statistical analysis

As patients usually seek treatment when their health is worse than average, an alleviation of their illnesses can easily be mistaken for an effect from the initiation of treatment, although it only represents natural variability. This phenomenon is widely known as “regression to the mean” (RTM), first described by Galton [30]. Thus, RTM should be clearly distinguished from treatment. One possibility to do so was outlined by Mee and Chua [31] who proposed a modified t-test which allows to estimate a “treatment effect” taking into account that RTM might be present. In contrast to other approaches Mee and Chua’s procedure only requires the true mean μ in the target population to be known. As in the case of the tinnitus questionnaire this condition is not given, we used the mean of 251 patients screened in the Audiology department of the University Clinic of Münster provided in the TF-Manual [32] as a proxy for the global tinnitus score and all six subscales.

As the proxy for the global tinnitus score and the subscales might not be a optimal estimator for the “true” mean, we additionally followed the suggestions of Ostermann et al. [33] and varied μ systematically over a range of reasonable values, ran the Mee-Chua algorithm for each mean separately, and plotted the RTM adjusted effects and confidence intervals (CI) of the global tinnitus score against this mean. This gives an overall impression about how RTM affected the data.

All estimates are presented in two scales, first in the original scaling of the TF questionnaire, second as standardized effect estimates, which were obtained by dividing the estimate of the original scaling by the respective standard deviation at baseline. All descriptive statistics are given as mean ± standard deviation. Reported CIs are at a 95 % level.

## Results

### Sociodemographic data

Data of 5.536 outpatients treated between 2003 and 2010 at the tinnitus therapy center, Krefeld-Düsseldorf (TTZ) were included. Patients were mostly (83.9 %) of wage-earning years (i.e. between 18 and 65 years (Mean age: 49.8 ± 13.9 years), lived typically in a fixed partnership (69.1 %), were almost split half in male and female, and were usually employed as clerks (45.4 %) or not employed (33.9 %). Duration of tinnitus was positively skewed with a mean of 60.2 ± 82.5 months with 42,9 % of patients suffering from tinnitus for more than three years. Most disturbing for the patients according to their rating on a visual analogue scale (VAS: 0–10) was the disruption due to ear ringing (mean: 6.2 ± 2.4) followed by loudness (mean: 5.7 ± 2.1) and restrictions due to ear-ringing (mean: 4.7 ± 2.8). The complete data is provided in Table 1.

**Table 1:** Socio-demographic and anamnestic data (absolute numbers and percentages or mean ± standard deviation and median)

	Male	Female	Total	No answer
N	2.736 (49.9 %)	2.688 (49.0 %)	5.480	56 (1.0 %)
Age in years	49.8 ± 14.6; 50.0	49.8 ± 13.2; 50.0	49.8 ± 13.9; 50.0	59 (2.4 %)
Marital status				
▪ single ▪ Married/fixed partner ▪ Divorced/living separated ▪ Widowed	461 (16.9 %) 1.711 (62.5 %) 321 (11.7 %) 229 (8.4 %)	387 (14.4 %) 2.069 (77.0 %) 178 (6.6 %) 46 (1.7 %)	848 (15.5 %) 3.784 (69.1 %) 500 (9.1 %) 275 (5.0 %)	73 (1,3 %)
Graduation				
▪ Lower Secondary School ▪ Secondary School ▪ High-School ▪ University/College	885 (32.4 %) 863 (31.5 %) 529 (19.3 %) 416 (15.2 %)	985 (36.6 %) 677 (25.2 %) 393 (14.6 %) 589 (21.9 %)	1874 (34.2 %) 1.540 (28.1 %) 922 (16.8 %) 1.005 (18.3 %)	139 (2.5 %)
Profession				
▪ Work-men ▪ Clerk ▪ Self-employed ▪ Not employed	204 (7.4 %) 1.319 (48.2 %) 100 (3.6 %) 1.062 (38.8 %)	532 (19.8 %) 1.166 (43.4 %) 166 (6.2 %) 793 (29.5 %)	737 (13.4 %) 2.486 (45.4 %) 266 (4.9 %) 1.858 (33.9 %)	133 (2.4 %)
Duration of tinnitus in month	54.2 ± 75.5, 21.0	66.1 ± 88.5, 24.0	60.2 ± 82.5, 24.0	239 (9.6 %)
Loudness of ear-ringing (from 0 = „not at all“ to 10 = “maximum”)	5.8 ± 2.1, 6.0	5.7 ± 2.2, 6.0	5.7 ± 2.1, 6.0	278 (11.2 %)
Disruption due to of ear-ringing (from 0 = „not at all“ to 10 = “maximum”)	6.4 ± 2.5, 6.0	6.1 ± 2.4, 6.0	6.2 ± 2.4, 6.0	251 (10.1 %)
Restrictions due to ear-ringing (from 0 = „not at all“ to 10 = “maximum”)	4.8 ± 2.8, 5.0	4.5 ± 2.7, 4.0	4.7 ± 2.8, 5.0	278 (11.2 %)

### Outcomes

After 9 days of tinnitus training a reduction of −18.6 points (CI: −18.9 to –18.2, *p <* 0.001) of the TF-Score was estimated for all patients, which equals a high standardised effect of -d = −1.03 (CI: −1.05 to −1.01). Interestingly, men had a slightly higher benefit (−19.3 points corresponding to d = −1.09 (CI: −1.05 to −1.01)) compared to women (−17.8 points, d = −0.96 (CI: −1.05 to −1.01)). After 6 months a reduction of 16.9 points was estimated for all patients (d = −0.93; (CI: −1.05 to −1.01)). Again male patients showed a slightly higher reduction of tinnitus burden than women (−17.3 vs. -16.5 resp. d = −0.98 (CI: −1.05 to −1.01) vs −0.89 (CI: −1.05 to −1.01)). Due to multiple imputation 95 % confidence intervals were quite high in the follow up, however effects remained significant (Table 2).

**Table 2:** Estimated treatment effects adjusted for regression-to-the-mean (in brackets: 95 % confidence intervals) (*n =* 5.421)

	Treatment effect (RTM adjusted)		Standardised effect^a^ (RTM adjusted)		*p-*value
End of treatment					
All patients	−18.6	(−18.2 to −18.9)	−1.03	(−1.01 to −1.05)	<.0001
Men	−19.3	(−18.8 to −19.8)	−1.09	(−1.06 to −1.12)	<.0001
Women	−17.8	(−17.3 to −18.3)	−0.96	(−0.94 to −0.99)	<.0001
Follow-up					
All patients	−16.9	(−29.0 to −4.8)	−0.93	(−1.60 to −0.23)	0.0342
Men	−17.3	(−22.8 to −12.7)	−0.98	(−1.29 to −0.66)	0.0056
Women	−16.5	(−22.7 to −10.4)	−0.89	(−1.23 to −0.56)	0.0082

^a^Standardized effects: treatment effects divided by the standard deviations at baseline

Effects are based on the TQ total score

A more complex picture is drawn when looking at the TF subscales. While for the end of treatment, all subscales showed highly significant improvements with standardised effect sizes between –d = 0.31 for “somatic complaints” and d = −1.03 for “cognitive distress”, follow-up results were more heterogeneous: the scales “Emotional distress” and “Intrusiveness” still remained significant with d = −0.67 (CI: −1.05 to −1.01) and d = −0.90 (CI: −1.05 to −1.01) respectively. In contrast the subscales “Cognitive distress” and “Sleep disturbances” failed to reach significance although a trend for improvement after 6 month was visible (d = −0.60 (CI: −1.05 to −1.01) and d = −0.33 (CI: −1.05 to −1.01) resp.). Complete data is shown in Table 3.

**Table 3:** Estimated treatment effects adjusted for regression-to-the-mean (in brackets: 95 % confidence intervals) (*n =* 5.421)

	Mean	Treatment effect (RTM adjusted)	Standardised effect^a^ (RTM adjusted)	*p-*value
End of treatment				
Emotional distress	11.3	−5.9 (−6.1 to −5.8)	−1.00 (−1.02 to −0.97)	<.0001
Cognitive distress	8.0	−4.3 (−4.4 to −4.2)	−1.03 (−1.06 to −1.00)	<.0001
Intrusiveness	9.8	−3.7 (−3.8 to −3.6)	−0.98 (−1.01 to −0.96)	<.0001
Auditory perceptual difficulties	5.8	−2.4 (−2.5 to −2.3)	−0.61 (−0.63 to −0.59)	<.0001
Sleep disturbances	2.8	−1.1 (−1.2 to −1.0)	−0.41 (−0.43 to −0.38)	<.0001
Somatic complaints	1.9	−0.7 (−0.8 to −0.6)	−0.31 (−0.34 to −0.28)	<.0001
Follow-up				
Emotional distress	11.3	−4.2 (−5.2 to −2.3)	−0.67 (−0.96 to −0.39)	<.0001
Cognitive distress	8.0	−2.5 (−7.9 to +2.9)	−0.60 (−1.90 to +0.70)	0.1126
Intrusiveness	9.8	−3.4 (−4.1 to −2.7)	−0.90 (−1.09 to −0.72)	<.0001
Auditory perceptual difficulties	5.8	−0.1 (−2.2 to +2.1)	−0.03 (−0.56 to +0.53)	0.9273
Sleep disturbances	2.8	−0.9 (−2.0 to +0.2)	−0.33 (−0.72 to +0.07)	0.0702
Somatic complaints	1.9	−0.4 (−2.8 to +2.0)	−0.17 (−1.27 to +0.94)	0.4013

^a^ Standardized effects: treatment effects divided by the standard deviations at baseline

**Fig. 2:**
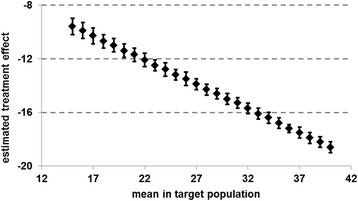
Estimated treatment effects adjusted for regression-to-the-mean at the end of treatment for various assumptions on the mean in the target population (error bars show 95 % confidence intervals) (*n =* 5.421)

**Fig. 3:**
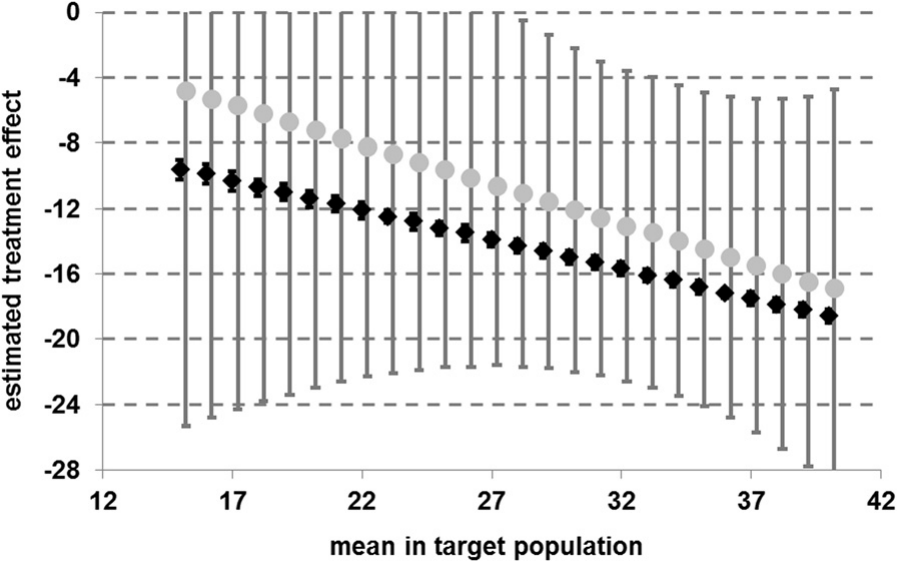
Estimated treatment effects adjusted for regression-to-the-mean at the end of treatment (black squares) and at follow-up (grey cirlces) for various assumptions on the mean in the target population (error bars show 95 % confidence intervals) (*n =* 5.421)

As already mentioned the estimated treatment success sensitively depends on the value of the μ representing the mean TF-score in the target population. This however is not known and thus was approximated in our analysis so far. Figure 2 provides the RTM-adjusted treatment effects for a wide range of values for μ. At the end of treatment treatment effects of more than 12 points can be assumed if μ takes values greater than 22 points. At follow-up, significant effects of more than 12 points reduction can be found for μ larger than 28 points. Figure 3 displays a comparison of effect sizes at the end of treatment and after follow-up which illustrates the difference in their variability. Still a significant effect for patients with mediocre tinnitus can be assumed.

## Discussion

This paper presents the results of the largest study of the effects of an outpatient tinnitus therapy program with a total of 5421 patients included in the final analysis. Based on multiple imputations and RTM control we not only were able to provide robust effect sizes for the end of treatment but also in a modelling approach for the follow-up of 6 month after end of therapy. In particular our data suggests that complex outpatient therapy as provided by the Tinnitus Center Krefeld-Duesseldorf leads to an immediate reduction of emotional and cognitive distress and intrusiveness related to tinnitus which according to our modelling approach is suspected to be stable in the follow up after 6 month. These results confirm findings that favour the multi-discipline approach for instance those by a study by Zöger et al. [34] in which *N =* 37 tinnitus patients participated in outpatient group psychotherapy. They found a reduction in anxiety in patients receiving elements of cognitive behavioural therapy (CBT) and a close association of emotional and physical arousal and tinnitus suffering. Similar results were observed by Sadlier et al. [35] in a small controlled study of 25 patients suffering from chronic tinnitus in Wales. They found that short term interventions with CBT and meditation techniques led to significant reductions in tinnitus burden. However, another RCT of mindfulness-based cognitive therapy in 30 tinnitus patients of Philippot et al. [36] did not did not find significant differences between pre- and post-treatment.

In the field of music therapy results from the Heidelberger model [37] found similar effects in an earlier observational pilot study of 23 patients with TF scores: declining from 40.1 ± 11.4 at baseline to 27.9 ± 12.8 after treatment and 24.0 ± 12.2 after three month follow-up. Another clinical trial of Davis et al. [38] on neuromonics tinnitus treatment (a pattern of acoustic stimuli designed to retrain the neural pathways) combining acoustic stimulation with counselling elements and support found statistically significant improvements in tinnitus distress and awareness in 35 patients with moderate-to-severe level of tinnitus-related distress.

These findings suggest that a single approach might not be as effective as a combined approach similar to what we applied in our research presented here. This is underpinned by several researchers who proposed the use of sound with educational elements and stress-reduction techniques supplemented with psychological and medical management as the most promising way to treat tinnitus [9, 39, 40].

### Limitations

There are certain limitations to our study. The imputation of a large percentage of data in our population due to missing values is certainly the most critical aspect that limits the generalizability of our results. Although the massive imputation of missing data does only give a theoretical framing of potential sustainability, studies in large data sets in the Children’s mental health initiative have shown the usability of multiple imputation in cases of missing values ranging up to 99 % [41]. However, further research into its performance parameters and limitations is needed.

The most obvious disadvantages of a cohort study are usually that they are expensive, time-consuming and inefficient for rare outcomes with long induction or latency periods, which is not the case in our study. Nevertheless, there is a higher risk for drawing incorrect inferences about treatment effects because of the increased likelihood of bias (because treatments are not randomly assigned); therefore, results from observational studies like ours should be confirmed by randomized trials whenever possible. In this respect trials like the already mentioned survey of Argstatter [13] is a good example.

In some respect our results are in accordance with those findings from a perspective of health service research: while most of the studies in the field of alternative therapies for tinnitus suffer from high numbers of patients [42], we were able show real world effectiveness. Although our approach suffers a lack of follow up data resulting in large confidence intervals in effect sizes, we nevertheless were able to provide high effect sizes adjusted for regression to the mean in more than 5400 patients.

## Conclusion

A great variety of models and treatment approaches are available for the treatment of tinnitus ranging from outpatient treatments by family physicians to long-term inpatient treatments in specialized rehabilitation hospitals. Our approach aims at filling the gap between these two approaches. Based on a close-to-home outpatient treatment, the patient stays in his/her social environment, which reduces the recurrence rate, which is often observed in patients treated in a secure environment of a hospital.

In line with the previously stated arguments, our data suggests that such a combined approach including musical, cognitive, behavioural and mindfulness elements is a promising way of treating chronic tinnitus patients [43]. As most of the patients are members of the statutory health insurance, future studies should try to complement already existing data with health economic parameters such as days of work absence, post treatment GP attendance due to tinnitus or simply the cumulative treatment costs before and after outpatient treatment in the Krefeld Model.

## Abbreviations

μ: Mean TF-score in the Target Population
AST®: Auditive Stimulation Therapy
CBT: Cognitive Behavioural Therapy
CI: Confidence Interval
d: Effect Size
MCMC: Markov Chain Monte Carlo
MI: Procedure of SAS 9.2 for multiple imputations
MiAnalyze: Procedure of SAS 9.2 for Multiple Imputations
RCT: Randomized controlled trial
RTM: Regression to the Mean
SAS: Statistical Analysis Software
Std-dev: Standard Deviation
TF: Tinnitus-Questionnaire
TTZ: Tinnitus-Therapy Center, Krefeld-Düsseldorf
